# Reduced Muscle Activity of the Upper Extremity in Individuals with Spinal Cord Injuries

**DOI:** 10.3390/ijerph19084708

**Published:** 2022-04-13

**Authors:** Kyung-Sun Lee, Mobasshira Zaman, Jaejin Hwang

**Affiliations:** 1 Division of Energy Resources Engineering and Industrial Engineering, Kangwon National University, Chuncheon-si 24341, Gangwon-do, Korea; kyungsunlee81@gmail.com; 2 Department of Industrial and Systems Engineering, Northern Illinois University, DeKalb, IL 60115, USA; z1934124@students.niu.edu

**Keywords:** SCI patient, muscle activity, EMG, spinal cord injury, upper extremity

## Abstract

Compromised physical ability due to musculoskeletal impairment among spinal cord injury (SCI) patients is known to negatively affect their quality of life. It is essential to comprehensively understand the muscle strength of the upper extremity among patients with SCI to enhance muscle function and capacity to engage in an active lifestyle. The objective of this study was to evaluate the muscle strength of 15 upper extremity muscles among patients with SCI and compare the relative weakness of individual muscles to the control group. Seven male patients with SCI with ASIA impairment scale D and E and 33 males in the control group participated in this study. Each participant performed maximal voluntary contraction of individual muscles, and the electromyography data were recorded. The results showed that the majority of the upper extremity muscles (12 out of 15) showed considerable weakness (24 to 53%) relative to the control group. Furthermore, the relative strength (ranking) of individual muscles among 15 upper extremity muscles was different between patients with SCI and the control group. This information would be useful to the selective strengthening of specific muscles as an intensive rehabilitation effort and prevent overuse and adverse injuries due to excessive muscle training.

## 1. Introduction

Spinal cord injury (SCI) is a catastrophic event that may result in permanent disability or loss of function in motion and posture [1,2]. In 2016, on average 27,000,000 people suffered from SCI globally [3], and according to the National Spinal Cord Injury Statistical Center, in 2021 only in the United States the prevalence of SCI was around 296,000 [4]. For patients with SCI in the United States military, age, sex, and race were significantly associated with the risk factors of SCI, and war-related SCIs revealed different characteristics compared to traumatic SCI among the general population [5,6]. In the United States, the total expenditure for spinal cord injury was approximately $14.5 billion; in 2019 dollars, the inflation rate was $23 billion per year [7]. SCI refers to abnormalities in motor, sensory, and autonomic nerve functions due to trauma to the spinal cord [8]. Although medicine continues to make remarkable progress in this area, it is still difficult to regenerate the damaged spinal cord. Only relatively recently was partial functional recovery successful in animal experiments, and there are reports that some functions have been restored in humans by cell transplantation, such as embryonic stem cell transplantation [9,10]. However, since most cases of spinal cord injury cause serious motor dysfunction, active rehabilitation treatment for functional improvement is absolutely necessary.

**Figure 1:**
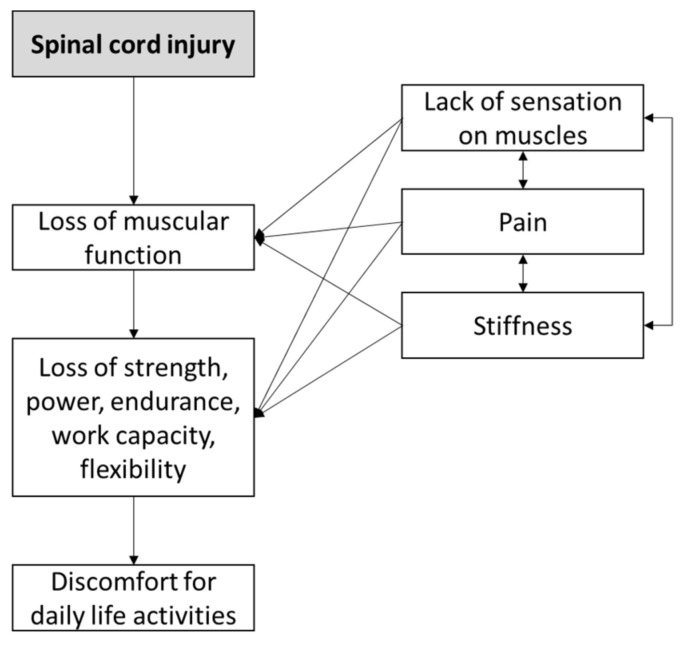
Muscular issues after spinal cord injury.

Patients with SCI are known to have a loss of muscular function compared to the healthy population [11]. Due to lack of sensation in muscles and sometimes pain associated with stiffness in muscle movements, spasms, and overall spasticity, the daily life activities of spinal cord–injured patients became more difficult, as seen in Figure 1 [12]. Lower maximum voluntary muscle contraction was common for injured area muscles, which leads to functionality constraints in daily work. Not only the spinal region and lower extremity, but the pain in the upper extremity also lead to discomfort for various daily life activities [13]. Patients with SCI showed a decreased upper extremity work capacity compared to healthy individuals [14,15]. In particular, patients with SCI have limited proficiency in the repeated tasks of daily living that require physical strength, power, and endurance. Compared with healthy individuals, patients with SCI had dramatically less upper body strength and a significantly reduced work capacity, limiting their independence and promoting inactivity [16]. Typically, the hands of SCI patients were paralyzed while some shoulder and elbow muscles were weak but retained some voluntary control [17].

Patients with SCI determine the degree of severity according to the loss of muscle function and the degree of pain [18]. There are several levels of SCI depending on the severity of the symptom, according to the American Spinal Injury Association (ASIA). Based on the motor function and sensory condition, the degree of impairment was leveled on five scales from A to E. Level A implies complete impairment, including both sensory and motor non-functionality, while levels B, C, and D indicate incomplete impairment based on the preservation of motor and sensory function. Level E stands for the patients with normal motor and sensory function with some reflex and neurological constraints [7].

Depending on the area of the spinal cord injury, cervical spine injury causes disturbances in body temperature control and upper arm functions, resulting in reduced exercise capacity. In addition, disabled persons with damage to the 6th thoracic vertebra or more have limited instrumental activity because they depend on a wheelchair for daily activities and movement [19]. In addition, health-related physical fitness and motor functions such as strength, muscular endurance, flexibility, and cardiorespiratory endurance are significantly reduced [20]. Changes in bodily functions due to spinal cord injury accompany many difficulties in the patient’s daily living movements and social activities depending on the part of the disability and the degree of damage. Since spinal cord injury patients have many restrictions in their daily life and in performing various tasks, it can be said that they are the main targets of rehabilitation [Donnelly et al.]. According to the study of [21], the ultimate goal of rehabilitation of spinal cord injury patients is walking, and the following sequence is required for upper extremity functions such as eating and feces and urine processing. The reason why the functional recovery of the upper extremities is so important is that it has a decisive influence on the degree of assistance required for daily activities and independent activities at home. Over the last few decades, various interventions have evolved in an attempt to improve upper limb function in individuals with SCI [22].

Numerous studies have been conducted to evaluate the importance of upper extremity rehabilitation and the effect of rehabilitation in patients with SCI [23,24,25]. However, there has been a lack of information on how the upper extremity muscle functions of patients with SCI are different from the healthy population. Accurately assessing the muscle activity of patients with SCI compared with the healthy population is very important in determining effective rehabilitation training and programs. It is also very important information in terms of preventing injuries that may occur due to excessive rehabilitation. The objective of this study was to evaluate the maximum voluntary contractions of the upper extremity muscles of patients with SCI and compare them with the healthy group. This investigation would help to understand what specific muscle groups are weakened among patients with SCI, and this would guide rehabilitation strategies.

## 2. Materials and Methods

### 2.1. Participants

With a confidence level of 95% and a minimum acceptable power of 80%, to find the difference in median values (50% relative difference) between the two groups, the required sample size was 17. A control group (33 male) and a group consisting of SCI patients (7 male), recruited via flyers posted on campus and in the surrounding community, participated in the experiment. In the control group, all participants were right-handed and had no history of illnesses or injuries. In the SCI group, all participants were right-handed and had ASIA impairment scale D (5 participants) and E (2 participants) levels. ASIA impairment scale D level defines that motor function is preserved below the neurological level; for this level, at least half of the key muscles below the neurological level have a muscle grade of 3 or more. ASIA impairment scale E level defines that motor and sensory function are normal.

All of the participants provided informed consent before participation according to the requirement of the university’s Institutional Review Board. The mean (standard deviation; SD) age, height, and weight of the participants were 29.5 (SD 5.2) years, 172.3 (SD 4.3) cm, and 68.1 (SD 19.2) kg, respectively. The mean (standard deviation) right-hand length, circumference, and breadth were 17.2 (SD 1.6) cm, 19.8 (SD 1.2) cm, and 9.1 (SD 1.2) cm, respectively.

### 2.2. Equipment

A Telemyo 2400 direct transmission system (DTS) (NORAXON, Scottsdale, AZ, USA) with 15 channels and disposable, self-adhesive Ag/AgCl dual-snap electrodes was used to record EMG signals. The figure-eight-shaped adhesive area was 4 × 2.2 cm^2^, the diameter of both circular conductive areas was 1 cm, and the inter-electrode distance was 2 cm. The EMG signals were amplified with a gain of 500. They had a noise of <1 µV and a sampling rate of 1000 Hz, with a filtering bandwidth of 20–450 Hz. All of the data were acquired on a laptop computer with a 16-bit analog-to-digital converter.

### 2.3. Experimental Procedures

**Figure 2:**
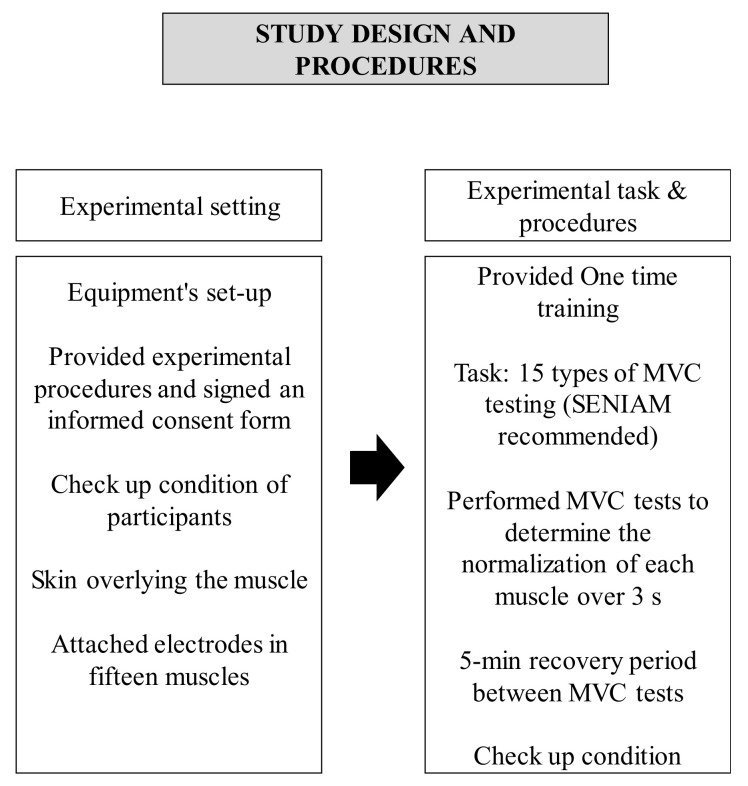
Study design and procedures.

Each participant was provided with a brief description of the experimental procedures and signed an informed consent form. The less-impaired side of patients with SCI and the dominant side of healthy participants were assessed. All control group participants were right-handed. For the SCI group, all participants except one had the right-upper extremity as the less-impaired side. Following the recommendations of SENIAM (surface EMG for noninvasive assessment of muscle) [26], the skin overlying the muscle was shaved and cleaned with alcohol and gauze to minimize impedance (Figure 2).

Dual-surface electrodes were used to record the activities in fifteen upper extremity surface muscles related to shoulder segment motion, elbow segment motion, wrist segment motion, and finger segment motion: anterior deltoid (AD), latissimus dorsi (LD), middle deltoid (MD), middle trapezius (MT), posterior deltoid (PD), brachioradialis (B), biceps brachii (BB), triceps brachii (TB), extensor carpi radialis (ECR), extensor carpi ulnaris (ECU), flexor carpi radialis (FCR), flexor carpi ulnaris (FCU), extensor digitorum comunis (EDC), flexor digitorum superficialis (FDS), and palmaris longus (PL). The electrode positions were chosen according to the recommendations of SENIAM and other studies [26,27,28,29].

After attachment of the electrodes, each participant performed maximal voluntary contraction (MVC) tests to determine the normalization of each muscle over 3 s (Table 1). There was a 5-min recovery period between MVC tests to avoid localized muscle fatigue. The order of muscle testing was randomized. The patients with SCI continuously checked whether the task was progressing before performing the experimental trials, and even after a 5-min rest period, an additional rest period was given if the patients wanted it. The SCI group used a wheelchair, and the control group sat on a chair to perform the task.

### 2.4. Data Analysis

The mean and standard deviations of mean, peak, and coefficient of variation (CV) were calculated for each muscle for the control and SCI groups. Since a normalization approach was difficult to conduct in this study, CV was analyzed to understand the variation in EMG of individual muscles of the participants. Furthermore, the percentage differences between the control and SCI groups for all 15 muscles were computed. Rankings of the peak muscle activities were summarized for each SCI and control group.

Due to the non-normality of the data, the Mann–Whitney non-parametric test was performed using SPSS software (IBM SPSS Statistics 26.0). The independent variable was the patient type (patients with SCI and control) and the dependent variables were the mean, peak, and CV for individual muscles. The statistical significance was set as 0.05.

## 3. Results

### 3.1. Shoulder Muscles

Table 2 shows the mean and SD of mean, peak, and CV of individual muscles associated with the shoulder joint between the control and SCI groups with *p*-values. The percentage difference relative to the control group was also summarized.

There was a significant difference in mean muscle activities of LD between the SCI and control groups (*p* = 0.018). The SCI group showed 53.69% lower mean muscle activities of LD compared to the control group. The SCI group revealed 37.44% and 19.30% lower peak muscle activities of MT and PD compared to the control group. There was a significant difference in CV for the MT (*p* = 0.034) muscle between the SCI and control groups. The patients with SCI showed 42.45% and 35.07% less CV of MT and PD compared to the control group, respectively. Although patients with SCI showed greater mean and peak values of AD muscle compared to the control group, there was no statistical difference (*p* > 0.803).

### 3.2. Elbow Muscles

Table 3 shows the mean and SD of mean, peak, and CV of individual muscles associated with the elbow joint between the control and SCI groups with *p*-values.

There were significant differences in mean and peak muscle activities of B and TB between the SCI and control groups (*p* < 0.015). The SCI group showed 40.40% and 25.69% lower peak muscle activities of B and TB compared to the control group. There was a significant difference in CV for the TB muscle between the SCI and control groups (*p* = 0.041). The SCI group showed 46.46% less CV of TB compared to the control group.

### 3.3. Wrist Muscles

Table 4 shows the mean and SD of mean, peak, and CV of individual muscles associated with the wrist joint between the SCI and control groups with *p*-values.

For the mean muscle activities, ECU and FCU showed significant differences between the SCI and control groups (*p* < 0.022). The SCI group showed 44.59% and 48.07% lower mean muscle activities of ECU and FCU compared to the control group. There were significant differences in peak muscle activities of ECU and FCU between the SCI and control groups (*p* < 0.022). The SCI group showed 46.73% and 52.70% lower peak muscle activities of ECU and FCU compared to the control group, respectively.

### 3.4. Finger Muscles

Table 5 shows the mean and SD of mean, peak, and CV of individual muscles associated with the finger joint between the SCI and control groups with *p*-values.

There were no significant differences in mean and peak muscle activities between the SCI and control groups (*p* > 0.094). The CV was also not significantly affected by the group (*p* > 0.171). Although patients with SCI showed higher mean and peak values of PL muscle relative to the control group, there was no statistical difference (*p* > 0.762).

### 3.5. Muscle Activity Rankings

**Figure 3:**
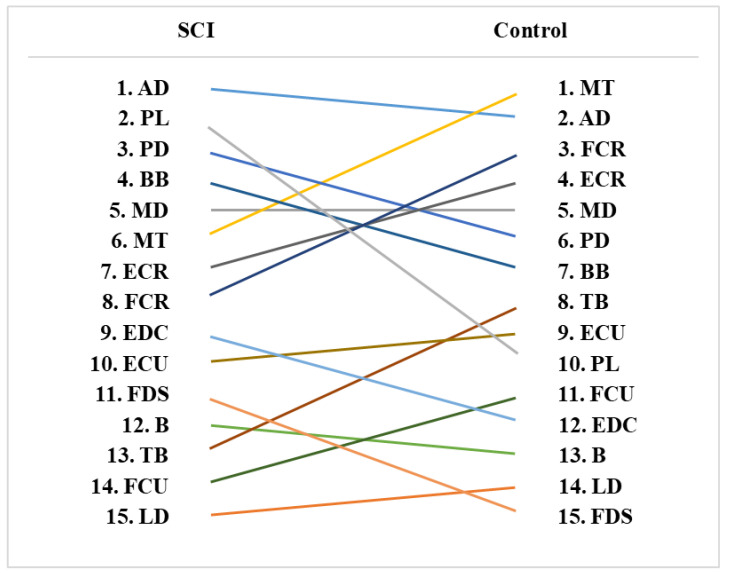
Muscle activity (peak value) rankings for the SCI and control groups.

Figure 3 shows the muscle activity (peak value) rankings for the SCI and control groups. There were differences in muscle activity rankings between the two groups. MT muscle activities were ranked 1 in the control group but 6 in the SCI group (percentage difference = 37.44%). The FCR muscle activities were ranked 3 in the control group but 8 in SCI group (percentage difference = 38.37%). The ECR muscle’s ranking was 4 in the control group but 7 in the SCI group (percentage difference = 35.27%).

## 4. Discussion

The compromised physical ability of patients with SCI is known to negatively affect their quality of life [30]. Musculoskeletal impairment is one of the main causes of the reduced ability in daily functions [31]. It is important to thoroughly understand the muscle strength of the upper extremity of patients with SCI to improve muscle function and capacity to engage in an active lifestyle. This study investigated the muscle activities of the 15 upper extremity muscles in the SCI and control groups while performing MVC tests. It was found that the majority of the upper extremity muscles (12 out of 15) showed considerable weakness (24 to 53%) relative to the control group. In addition, the relative strength (ranking) of individual muscles among 15 upper extremity muscles was different between the SCI and control groups.

For the muscles associated with the shoulder joint, LD showed the greatest difference (up to 54%) between the SCI and control groups. In addition, MD (up to 33%) and MT (up to 37%) showed substantial differences in muscle activities between the two groups. For MT, the control group exhibited greater variation (CV) of muscle activities among participants compared to the SCI group. A previous study also found that relative weakness in the shoulder adductors and internal rotators was found among the patients who were diagnosed with shoulder impingement syndrome [31]. Shoulder pain is known to be prevalent in patients with SCI, and Rotator cuff problems were frequently identified [31,32,33]. Previous studies showed that shoulder pain is primarily caused by an excessive overload on the upper limbs, such as wheelchair propulsion and transfers [32,34]. This overuse of the shoulder muscles could lead to degenerative conditions of the shoulders [33]. It was also found that muscle strength was inversely related to shoulder pain [35]. Therefore, the SCI patients recruited for this study were likely to experience chronic shoulder pain, which could reduce the muscle strength of their shoulders compared to the control group.

For the muscles related to the elbow joint, TB showed the highest reduction (up to 53%) in muscle activities in the SCI group compared to the control group. For TB, the control group revealed higher variation (CV) of muscle activities relative to the SCI group. Moreover, B (up to 40%) and BB (up to 26%) showed substantial differences in muscle activities between the two groups. This result was similar to the previous study that tested the proximal upper extremity muscles, including B and TB, of the participants in the SCI and control groups [36]. Based on a ranking analysis, asymmetric impairment of the elbow flexor (BB) and elbow extensor (TB) was observed in the SCI group. The ranking of BB was 7, whereas the ranking of TB was 13. In contrast, rankings of BB and TB were 7 and 8 among control group participants. Previous literature described that typically higher impairment was noted for the elbow extensor, and lower impairment was observed for the elbow flexor [37,38,39,40]. This was consistent with our study results.

For the muscles associated with the wrist joint, FCU (up to 53%) showed the most significant differences in muscle activities between the SCI and control groups. In addition, ECR (up to 35%), ECU (up to 47%), and FCR (up to 38%) showed the considerable weakness in muscle activities in the SCI group relative to the control group. In addition to shoulder pain, carpal tunnel syndrome (CTS) is another common upper extremity pain in SCI patients [31], one that has the potential to reduce muscle strength associated with the wrist.

In terms of the muscles related to the finger joint, EDC (up to 33%) showed the biggest reduction in muscle activities in the SCI group compared to the control group. Furthermore, FDS (up to 24%) showed a considerable difference in muscle activities between the two groups. Impaired hand function is known to reduce the functional capability and independence of daily activities [23].

For the muscle ranking comparisons between the SCI and control groups, 10 out of 15 muscles showed substantial differences in rankings between the two groups. For the control group, MT, FCR, and ECR were ranked 1, 3, and 4, whereas the same muscle was ranked 6, 7, and 8 for the SCI group. This indicates that relative muscle strength among upper extremity muscles is different between the two groups. The muscles associated with the shoulder and wrists were highly ranked in the control group, while the muscles related to the wrist were middle- or low-ranked in the SCI group.

Although this study was carefully designed and controlled, several limitations were noted. First, we have focused only on patients with the D and E levels in the ASIA impairment scale. Our study findings could not be generalized to other levels, such as A, B, and C. In addition, we did not distinguish between the ASIA D and E levels for statistical analysis due to the low sample size. Depending on the severity of the impairment levels, the characteristics of the muscle activation and strength could change [31]. The effect of different levels of SCI (e.g., C5) on muscular function was not examined due to the low sample size of the SCI patients. Future studies could recruit more SCI patients as a function of different ASIA impairment scale levels and the local area of the SCI. Second, the number of patients with SCI recruited in this study was small compared to the control group. Due to the low sample size, it would be difficult to generalize our findings. Future studies could increase the number of subjects with SCI and match this with the number of control subjects. Third, only male participants were recruited in this study. Since SCI is not a condition that occurs only in males, future studies could recruit and assess both males and females. Fourth, our study results were based purely on EMG results. Functional assessments, including strength and skill, and the amount of shoulder pain, would be important to provide a comprehensive motor assessment of the upper extremity muscles. Fifth, the present study focused only on isometric muscle exertions under the controlled setting, such as MVC tests. Future studies could investigate the muscle activities and functional outcomes of daily living activities. Sixth, muscle activities were not normalized by each subject in this study. Instead, the coefficient of variation of muscle activities was assessed to understand the variation difference across subjects. Future studies could explore a way to normalize the muscle activities during the MVC test to facilitate a fair comparison of muscle activities across subjects. Lastly, the Zancolli classification hand function scale was not considered in this study. The association between hand function and muscular strength would be an important topic in future studies.

## 5. Conclusions

Improvement in upper extremity function is one of the primary needs of patients with SCI [23]. In order to effectively improve and optimize the functional ability of the upper extremity of SCI patients through an intensive rehabilitation program, a detailed understanding of muscle strength relative to the control group is needed. The present study showed that the majority of upper extremity muscles (12 out of 15) of SCI patients showed reduced peak muscle strength (24 to 53%) relative to the control group. The TB showed the highest reduction in peak muscle activities (53.0%), followed by FCU (52.7%), ECU (46.7%), LD (42.3%), and B (40.4%). This information would be useful to focus on the selective strengthening of specific muscles for an intensive rehabilitation effort and prevent overtreatment and adverse injuries due to excessive muscle training.

## Figures and Tables

**Table 1:** Description of MVC methods.

Joint	Muscles	Manual Muscle Testing
Shoulder	Anterior Deltoid (AD)	Shoulder abduction in slight flexion, with the humerus in slight lateral rotation. In the erect sitting position, it was necessary to place the humerus in slight lateral rotation to increase the effect of gravity on the anterior fibers.
	Latissimus Dorsi (LD)	Adduction of the arm, with extension, in the medially rotated position.
	Middle deltoid (MD)	Adduction of the scapula, with upward rotation (lateral rotation of the inferior angle) and without elevation of the shoulder girdle. The test position was obtained by placing the shoulder in 90° abduction and in lateral rotation sufficient to bring the scapula into lateral rotation of the inferior angle.
	Middle Trapezius (MT)	Shoulder abduction without rotation. When placing the shoulder in a test position, the elbow should be flexed to indicate the neutral position of the rotation.
	Posterior Deltoid (PD)	Shoulder abduction in slight extension, with the humerus in slight medial rotation. In the erect sitting position, it was necessary to place the humerus in slight medial rotation to have the posterior fibers in an antigravity position.
Elbow	Brachioradialis (B)	Flexion of the elbow, with the forearm neutral between pronation and supination. The belly of the brachioradialis must be seen and felt during this test because the movement can also be produced by other muscles that flex the elbow.
	Biceps Brachii (BB)	Elbow flexion slightly less than or at a right angle, with the forearm in supination.
	Triceps Brachii (TB)	Extension of the elbow joint (to slightly less than full extension).
Wrist	Extensor carpi Radialis (ECR)	Extension of the wrist toward the radial side. Elbow flexion makes the extensor carpi radialis longus less effective by placing it in a shortened position.
	Extensor Carpi Ulnaris (ECU)	Extension of the wrist toward the ulnar side.
	Flexor Carpi Radialis (FCR)	Flexion of the wrist toward the radial side
	Flexor Carpi Ulnaris (FCU)	Flexion of the wrist toward the radial side.
Finger	Extensor Digitorum Comunis (EDC)	Extension of the metacarpophalangeal joints of the second through fifth digits with the interphalangeal joints relaxed.
	Flexor Digitorum Superficialis (FDS)	Flexion of the proximal interphalangeal joint, with the distal interphalangeal joint extended, of the second, third, fourth, and fifth digits.
	Palmaris Longus (PL)	Tensing of the palmar fascia by strongly cupping the palm of the hand and flexing the wrist.

**Table 2:** Descriptive statistics and *p*-values of individual muscles associated with the shoulder joint between the SCI group and the control group.

Muscle	Variable	SCI Patient	Control	% Difference	*p*-Value
Anterior Deltoid (AD)	Mean (uV)	524.00 (282.00)	509.60 (267.90)	2.83	0.817
	Peak (uV)	657.00 (383.00)	640.10 (322.80)	2.64	0.803
	CV (%)	12.29 (5.37)	16.72 (10.94)	−26.50	0.510
Latissimus Dorsi (LD)	Mean (uV)	154.20 (74.50)	333.00 (249.70)	−53.69	0.018 *
	Peak (uV)	236.10 (111.50)	409.40 (294.10)	−42.33	0.113
	CV (%)	34.70 (47.60)	14.79 (8.26)	134.62	0.533
Middle deltoid (MD)	Mean (uV)	309.60 (122.10)	459.30 (237.20)	−32.59	0.140
	Peak (uV)	409.00 (158.20)	580.00 (291.40)	−29.48	0.182
	CV (%)	20.21 (20.88)	16.82 (9.71)	20.15	0.510
Middle Trapezius (MT)	Mean (uV)	329.60 (234.50)	497.00 (318.20)	−33.68	0.182
	Peak (uV)	404.00 (288.00)	645.80 (391.10)	−37.44	0.072
	CV (%)	11.79 (1.87)	20.49 (16.54)	−42.45	0.034 *
Posterior Deltoid (PD)	Mean (uV)	381.70 (164.00)	440.50 (140.30)	−13.35	0.153
	Peak (uV)	453.30 (203.90)	561.70 (168.20)	−19.30	0.085
	CV (%)	10.85 (4.82)	16.71 (8.66)	−35.07	0.079

Note: *: *p* < 0.05.

**Table 3:** Descriptive statistics and *p*-values of individual muscles associated with the elbow joint between the SCI group and the control group.

Muscle	Variable	SCI Patient	Control	% Difference	*p*-Value
Brachioradialis (B)	Mean (uV)	210.10 (83.20)	331.80 (132.00)	−36.68	0.015 *
	Peak (uV)	249.60 (102.70)	418.80 (169.00)	−40.40	0.011 *
	CV (%)	9.79 (2.97)	15.89 (13.17)	−38.39	0.149
Biceps Brachii (BB)	Mean (uV)	336.70 (207.20)	428.80 (273.40)	−21.48	0.332
	Peak (uV)	412.00 (258.80)	554.40 (356.40)	−25.69	0.242
	CV (%)	11.88 (2.86)	17.34 (11.81)	−31.49	0.487
Triceps Brachii (TB)	Mean (uV)	208.70 (60.00)	414.30 (255.30)	−49.63	0.015 *
	Peak (uV)	248.30 (69.60)	528.20 (322.40)	−52.99	0.008 *
	CV (%)	9.98 (1.90)	18.64 (14.39)	−46.46	0.041 *

Note: *: *p* < 0.05.

**Table 4:** Descriptive statistics and *p*-values of individual muscles associated with the wrist joint between the SCI group and the control group.

Muscle	Variable	SCI Patient	Control	% Difference	*p*-Value
Extensor carpi Radialis (ECR)	Mean (uV)	310.50 (211.40)	468.40 (230.80)	−33.71	0.122
	Peak (uV)	375.90 (250.40)	580.70 (287.00)	−35.27	0.098
	CV (%)	16.57 (12.34)	15.06 (10.11)	10.03	0.789
Extensor Carpi Ulnaris (ECU)	Mean (uV)	233.40 (149.70)	421.20 (237.40)	−44.59	0.022 *
	Peak (uV)	275.80 (160.70)	517.70 (286.90)	−46.73	0.018 *
	CV (%)	14.07 (13.83)	15.24 (12.70)	−7.68	0.423
Flexor Carpi Radialis (FCR)	Mean (uV)	309.10 (159.30)	479.00 (257.50)	−35.47	0.117
	Peak (uV)	373.40 (208.00)	605.90 (330.60)	−38.37	0.113
	CV (%)	10.77 (6.59)	16.13 (12.71)	−33.23	0.171
Flexor Carpi Ulnaris (FCU)	Mean (uV)	207.60 (106.90)	399.80 (241.60)	−48.07	0.015 *
	Peak (uV)	238.10 (122.10)	503.40 (308.20)	−52.70	0.009 *
	CV (%)	8.87 (2.47)	15.96 (14.37)	−44.45	0.098

Note: *: *p* < 0.05.

**Table 5:** Descriptive statistics and *p*-values of individual muscles associated with the finger joint between the SCI group and the control group.

Muscle	Variable	SCI Patient	Control	% Difference	*p*-Value
Extensor Digitorum Comunis (EDC)	Mean (uV)	250.80 (150.40)	350.70 (192.30)	−28.49	0.160
	Peak (uV)	290.60 (160.30)	433.20 (220.50)	−32.92	0.094
	CV (%)	10.28 (4.78)	16.55 (12.41)	−37.89	0.219
Flexor Digitorum Superficialis (FDS)	Mean (uV)	224.30 (119.50)	267.40 (121.60)	−16.12	0.583
	Peak (uV)	256.60 (122.30)	337.60 (156.10)	−23.99	0.332
	CV (%)	12.41 (11.53)	14.98 (14.92)	−17.16	0.306
Palmaris Longus (PL)	Mean (uV)	428.00 (461.00)	380.50 (257.00)	12.48	0.929
	Peak (uV)	528.00 (552.00)	508.90 (323.10)	3.75	0.762
	CV (%)	17.84 (13.38)	22.60 (16.38)	−21.06	0.171

## Data Availability

Not applicable.
